# Topographic analysis of pancreatic cancer by TMA and digital spatial profiling reveals biological complexity with potential therapeutic implications

**DOI:** 10.1038/s41598-024-62031-0

**Published:** 2024-05-18

**Authors:** Victoria Bingham, Louise Harewood, Stephen McQuaid, Stephanie G. Craig, Julia F. Revolta, Chang S. Kim, Shambhavi Srivastava, Javier Quezada-Marín, Matthew P. Humphries, Manuel Salto-Tellez

**Affiliations:** 1 Precision Medicine Centre of Excellence, The Patrick G Johnston Centre for Cancer Research, Queen’s University Belfast, Belfast, Northern Ireland BT9 7AE UK; 2 Cellular Pathology, Belfast Health and Social Care Trust, Belfast, Northern Ireland UK; 3 Leeds Teaching Hospitals NHS Trust, Leeds, LS9 7TF UK; 4 University of Leeds, St James’ University Hospital, Leeds, UK; 5 Division of Molecular Pathology, The Institute for Cancer Research, London, UK

**Keywords:** Pancreatic ductal adenocarcinoma, Topographic tissue microarrays, Image analysis, Biomarkers, Tumour heterogeneity, Digital spatial profiling, Cancer, Molecular biology

## Abstract

Pancreatic ductal adenocarcinoma (PDAC) remains one of the most lethal human malignancies. Tissue microarrays (TMA) are an established method of high throughput biomarker interrogation in tissues but may not capture histological features of cancer with potential biological relevance. Topographic TMAs (T-TMAs) representing pathophysiological hallmarks of cancer were constructed from representative, retrospective PDAC diagnostic material, including 72 individual core tissue samples. The T-TMA was interrogated with tissue hybridization-based experiments to confirm the accuracy of the topographic sampling, expression of pro-tumourigenic and immune mediators of cancer, totalling more than 750 individual biomarker analyses. A custom designed Next Generation Sequencing (NGS) panel and a spatial distribution-specific transcriptomic evaluation were also employed. The morphological choice of the pathophysiological hallmarks of cancer was confirmed by protein-specific expression. Quantitative analysis identified topography-specific patterns of expression in the IDO/TGF-β axis; with a heterogeneous relationship of inflammation and desmoplasia across hallmark areas and a general but variable protein and gene expression of c-MET. NGS results highlighted underlying genetic heterogeneity within samples, which may have a confounding influence on the expression of a particular biomarker. T-TMAs, integrated with quantitative biomarker digital scoring, are useful tools to identify hallmark specific expression of biomarkers in pancreatic cancer.

## Introduction

Pancreatic ductal adenocarcinoma (PDAC) remains one of the most lethal human malignancies. Despite progress in diagnosis and treatment of PDAC, the post-operative 5-year survival rate remains low. Indeed, most patients at the time of diagnosis present with distant metastasis or life-threatening complications derived from the disease^1^. The promise of personalised medicine, which has transformed the way we approach many common cancers, has not significantly altered the prognosis of PDAC. Recently, the use of an indolamine-2,3-dioxygenase (IDO-1) inhibitor in a nanovesicle-driven delivery fashion induced measurable and efficient anti-PDAC immunity^2^. The transforming growth factor β (TGF-β) signalling pathway is a general regulator of many cancer-related processes; in particular, SMAD4, a mediator of TGF-β signalling, is inactivated in over half of PDAC^3^. The combined production of IDO and TGF-β induces regulatory T-cells^4^. Additionally, it has been described that the pancreatic stellate cells produce the collagenous stroma of PDAC and interact with cancer cells to facilitate disease progression, with the hepatocyte growth factor (HGF)/c-MET axis representing a candidate pathway for this^5^. Studies suggest that a combined approach, targeting tumour cells by chemotherapy while inhibiting this specific pathway may represent a novel therapeutic strategy to improve outcomes in PDAC^6^.

Many of the histological features of PDAC explain its aggressive behaviour. For example poor histological differentiation, an extensive desmoplastic reaction, abundant lymphatic/vascular invasion and a tendency for perineural invasion are notorious in PDAC^7^. These illustrate many of the pathophysiological hallmarks of cancer classically described^8^.

A cancer with this poor clinical outcome requires new models for research interrogation. Tissue microarrays (TMA) are arguably the most established method of biomarker analysis in tissues^9–11^; recently, we have shown validity in the analysis of adaptive immunity and immune checkpoints in cancer when coupled with efficient digital pathology tools in TMAs^12–18^. Hereby we present a TMA targeted model of construction (topographic TMAs or T-TMAs), capturing cores from many histological hallmarks of cancer: (1) malignant epithelial-rich areas, (2) desmoplastic reaction, (3) peri-neural invasion, (4) peri-vascular extension, (5) tumour margin (epithelial-mesenchymal transition), (6) lymphocytic inflammation and (7) intravascular invasion, when obvious. We discuss the accuracy of the model to interrogate such tissue-based pathophysiological processes and we analyse, in the different patients and topographies using an objective digital pathology scoring approach when relevant: (a) the expression of SMA and T-cell infiltration (CD3); (b) the expression of IDO/TGF-β; and (c) c-MET expression by immunohistochemistry and RNA-ISH. Furthermore, we have used a Nanostring Digital Spatial Profiling approach, applied to individual TMA cores, to further emphasise gene expression differences that exist in different topographical regions. Overall, our results capture a very clear topography-specific expression, enabling observations of biomarker relationships that may be important in future therapeutic strategies.

## Materials and methods

### TMA construction and immunohistochemistry

Representative formalin-fixed, paraffin-embedded tissue samples were derived from 12 resected PDAC, accessed through the Northern Ireland Biobank^19^. H&E stained slides from each case was reviewed and donor blocks selected which contained the pathophysiological hallmarks outlined below. Sections were digitally scanned on an Aperio AT2 at × 40 magnification and scans transferred to a fully automated tissue microarrayer (TMA Grandmaster, 3DHISTECH (Budapest)). H&Es were digitally annotated by consultant clinical diagnosticians (MST and SMcQ) to select the distinct regions depicting the seven pathophysiological hallmarks described. Briefly, H&E image were presented on screen within the 3DHITECH CaseViewer software. TMA core annotations were digitally placed on areas of interest on the H&E images. Area selection was confirmed by consensus. From each of the 12 cases, up to 7 selected 1.0 mm cores were placed into a recipient TMA block and sections prepared for analysis, representing a total of 72 tissue cores.

Fully automated immunohistochemical analysis was performed on a Ventana BenchMark with Ultraview or Optiview DAB detection or on a Leica Bond RX with DAB polymer detection, following previously published methodologies^13–18^. Primary antibodies employed were Cytokeratin (AE1/AE3), as a marker of epithelial tumour presence and volume; CD3 (2GV6) as indicative of T-cell infiltration; CD31 (JC70A) to prove the presence of vascular-associated endothelium; SMA (ASM-1) to highlight the desmoplastic reaction; S100ß to expose neural structures; the immune checkpoint indoleamine-pyrrole 2,3-dioxygenase, IDO-1 (D5J4E) and c-Met (SP44). TGF-β is a global cytokine and, as such, detection by IHC is unreliable; therefore automated RNA-ISH for TGF-β (nts. 170-1649), and for c-Met (nts. 175-6505) was also performed to standard in-house protocols^20^.

To establish the accuracy of the TMA-targeted model to capture focal pathophysiological hallmarks of cancer, we compared the intended purpose of each TMA core against the histological or immunohistochemical evidence by direct analysis by two experts (MST & SMcQ). T-cell density (CD3) in individual cores and the relationship between desmoplasia (SMA) were quantified using HALO image analysis v2.2. IDO-1 and TGF-β were assessed microscopically. Image analysis quantification was conducted on c-Met protein with Definiens Architect v2.7 and for mRNA expression using HALO v2.2

### Next generation sequencing

H&Es from representative tumour and normal blocks from each case was annotated to enrich for tumour or normal epithelial content. 4 × 10 µm thick sections from each block were macrodissected and DNA extracted with a Promega Maxwell 16 FFPE tissue LEV DNA extraction kit on a Maxwell 16 instrument. DNA was quantified via Qubit (ThermoFisher) and QC performed using the Agilent 4200 Tapestation. NGS libraries were prepared and hybridisation-based target enrichment performed using the Roche HyperCap workflow and associated kits. For determination of mutational status a custom designed Next Generation Sequencing (NGS) panel (Roche SeqCap EZ) to detect single nucleotide variants (SNVs) in genes was employed, including *BRAF, CDKN2A, KRAS* and *TP53* which are frequently mutated in solid tumours (see supplementary table S1 for full panel details). This panel and kit were validated in-house and the final library was sequenced on a NextSeq 500 instrument using a Mid Output v2.5 (150 cycles) cartridge. The sequenced samples were then demulitplexed using Illumina’s bcl2fastq (v2.20.0.422). The generated fastq files were then aligned to human reference genome (v GRCh38/hg38) using Burrow-Wheeler Aligner (bwa, v0.7.17)^21^ following which Samtools (v4.0.12.0)^22^ was used for sorting, merging and filtering the bam files generated by bwa. GATK4’s (v 4.0.12)^23^ picard was used to sort, mark and remove the duplicated reads. GATK4 was also used for local realignment of reads around INDELs and base recalibration. Single nucleotide variants (SNVs) were detected against matched normal samples using GATK4 Mutect2^24^. GATK4 FilterMutectCalls was used for filtering the called variants and SNPEff^25^ was used to annotate the final filtered calls.

### Nanostring digital spatial profiling

3 × 4 µm sections were cut from the PDAC TMA block onto the centre of positively charged Superfrost Plus Micro slides. Sections were air-dried at room temperature overnight and shipped with dessicant to Nanostring (Nanostring Technologies, 530 Fairview Avenue Norh, Seattle, W 98109, USA). Nanostring technologies then performed Nanostring Digital Spatial Profiling (DSP) via their Technology Access programme (TAP). This procedure allows the accurate selection of regions of tissue based on protein stains and subsequent RNA extraction and sequencing of cells from those regions. Two different protein stains pan-cytokeratin (panCK) and the perineural marker S100β were used to drive the selection of Area of Illumination (AOIs) within and across the cores of our TMA. This allowed us to determine three different regions: (1) regions positive for panCK to highlight tumour; (2) regions positive for S100β to indicate perineural involvement adjacent to tumour; (3) other stromal regions negative for both panCK and S100β, according to established methodology^26^. Initial quality control of the RNA hybridization data demonstrated the experiment was successful and that RNA was well preserved in all TMA cores. 14,000 genes were above the Limits of Quantitation and available for analysis.

### Ethics approval and consent to participate

All procedures performed in studies involving human participants were in accordance with the ethical standards of the institutional and/or national research committee and with the 1964 Helsinki declaration and its later amendments or comparable ethical standards. Samples in this study (NIB Study Ref: NIB15-0168) were provided by the Northern Ireland Biobank. The Northern Ireland Biobank is a HTA Licenced Research Tissue Bank with generic ethical approval from The Office of Research Ethics Committee Northern Ireland (ORECNI REF 21/NI/0019) and can confer ethical approval for projects which have received human tissue from the bank. Corresponding tumour slides and blocks were retrieved and collated via the Northern Ireland Biobank. In accordance with the Human Tissue Act 2004, consent is not required for use of archived, de-identified tissue in research studies with ethical approval^42^.

## Results

### Topographic analysis

**Figure 1:**
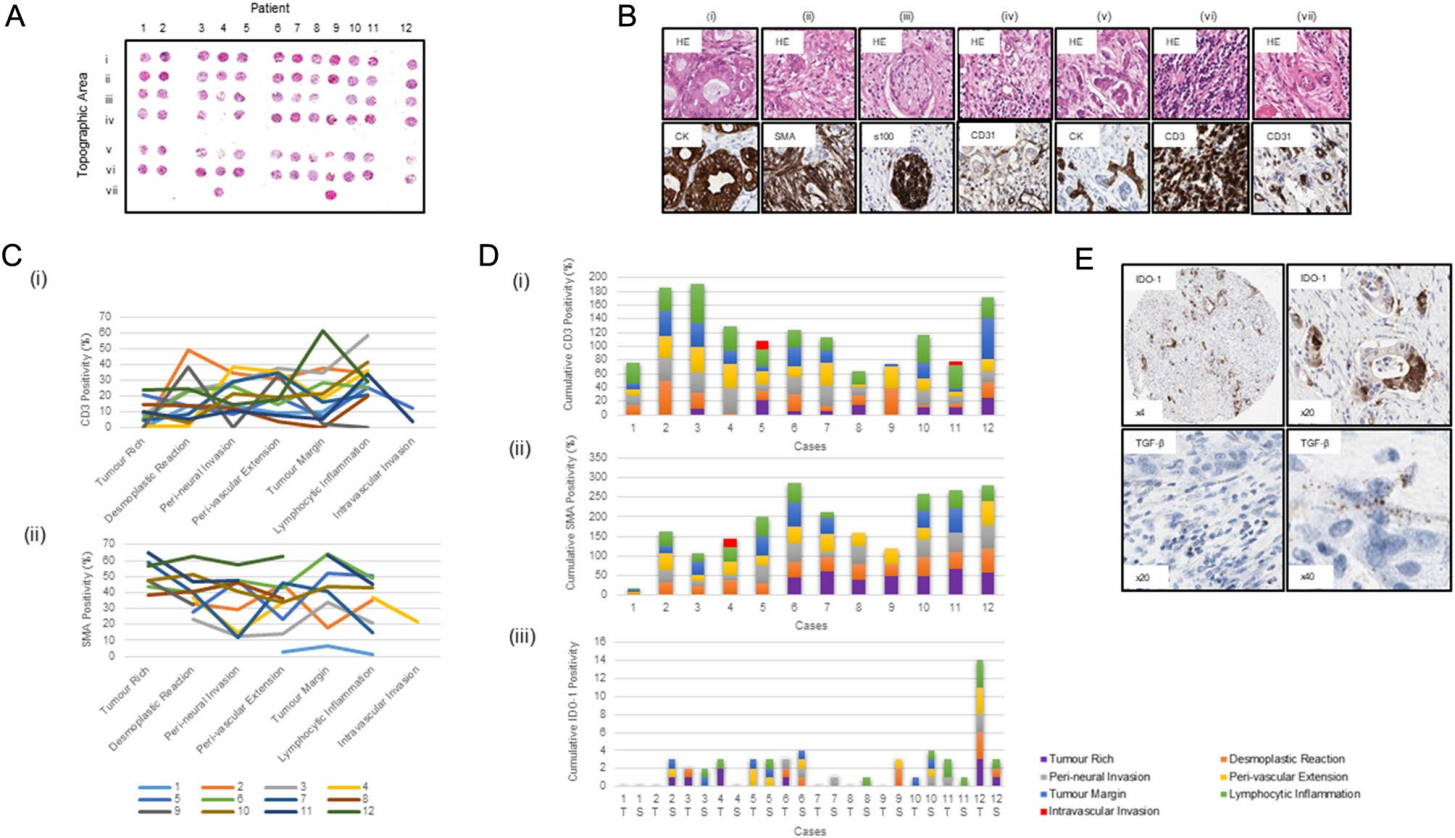
(**A**) H&E of TMA design from 12 PDAC blocks from which the described regions were selectively cored into a single recipient block. (i) Tumour rich, (ii) desmoplastic reaction, (iii) peri-neural invasion, (iv) peri-vascular extension, (v) tumour margin (epithelial-mesenchymal transition), (vi) lymphocytic inflammation and (vii) intravascular invasion, when obvious. (**B**) H&E and relevant immune markers to describe the accuracy of the TMA annotations (i–vii, as described above). (**C**) Line graph displaying the range of CD3 (i) and SMA (ii) in each of the topographical areas for cases 1–12. (**D**)(i) bar chart displaying the cumulative CD3 percentage positivity for cases 1–12 encompassing the topographic areas where data was available, with SMA data shown in (ii). (**D**)(iii) Expression of IDO1 in the tumour and stromal compartments, in different topographic areas for cases 1–12, T, tumour; S, stroma. 1E, focal IDO-1 IHC expression in a TMA core at × 4 and × 20 and TGF-β mRNA expression by RNA-ISH shown at × 20 and × 40.

Figure 1A describes the design of the pancreatic TMA. All the proposed topographic areas were arrayed in 12 cases, with 2/12 displaying additional definite regions of intravascular tumour as shown (Fig. 1Avii 4 and 9). Figure 1B illustrates a representative area rich in: tumour (i), desmoplastic reaction (ii), peri-neural invasion (iii), peri-vascular extension (iv), tumour margin (epithelial-mesenchymal transition) (v), lymphocytic inflammation (vi), and intravascular invasion when obvious (vii). The transfer of each pathophysiological region of interest to the TMA was highly accurate, as confirmed by visual pathological identification of the histological structures or processes and by immunohistochemical detection.

Figure 1C demonstrates the wide heterogeneity of immune reaction (CD3) (i) and desmoplasia (SMA) (ii) in each topographical area as measured quantitatively using HALO image analysis v2.2. These data are further represented at the individual case level in supplementary figs. S1 and S2, respectively. Figure 1D shows the range of T-cell density (CD3) (i), desmoplastic reaction (SMA) (ii) and IDO1 expression (iii) in the different topographical areas. Complete data for all topographical areas was available in cases, 3–7 and 10–12 for CD3, in cases 6, 7 and 10 for SMA and in cases 1–2 and 5–11 for IDO1. CD3 expression varied to a greater degree across the cases while SMA displayed a more consistent positivity quantitatively.

Analysis also provided early evidence of two interesting observations about IDO-1 and TGF-β mRNA. Focal expression of IDO-1 on tumour epithelial cells was seen in 7/12 patients but would only have been 3/12 cases if only tumour rich cores had been sampled. In case 10 IDO1 expression was only seen on a small number of tumour epithelial cells at the tumour margin and in case 11 IDO1 expression was only observed on tumour cells in TMA cores taken from peri-neural and lymphocytic rich regions (Fig. 1D iii).

In only 2/12 cases was expression of TGF-β mRNA observed and only on 2 of the 7 TMA cores in each case. Expression was of low or moderate intensity when compared with hybridisation-specific house-keeping genes (Fig. 1E). Such low level expression of TGF-β mRNA would not have been observed if only epithelial rich tumour regions had been sampled. We also observe that the only cases positive for TGF-β were indeed the cases with the highest desmoplastic reaction (Table 1).

**Table 1:** Comparison of specific tumour mutations with the IHC or RNA-ISH score of biomarkers known to influence tumorigenesis.

Case no.	KRAS	DDR2	PMS2	TP53	FBXW7	H3F3C	CDKN2A	MYCN	BARD1	RAD51C	PTEN	Mutation no.	Desmoplastic reaction (SMA)	T-cell density (CD3)	IDO-1 IHC Score	TGFb mRNA score	c-Met IHC H score	c-Met RNAScope score (ACD)
1	WT	WT	WT	WT	WT	WT	WT	WT	WT	WT	WT	0	4.8	30	−ve	−ve	30	1.0
2	MT	MT	WT	WT	WT	WT	WT	WT	WT	WT	WT	2	33.1	49	+	−ve	90	3.0
3	WT	WT	MT	WT	WT	WT	WT	WT	WT	WT	WT	1	23.2	58	+	−ve	140	1.6
4	MT	WT	WT	MT	WT	WT	WT	WT	WT	WT	WT	2	37.1	38	++	−ve	50	0.8
5	MT	WT	WT	MT	WT	WT	WT	WT	WT	WT	WT	2	27.9	26	−ve	−ve	140	2.5
6	MT	WT	WT	MT	WT	WT	WT	WT	WT	WT	WT	2	39.9	26	+	−ve	180	3.2
7	MT	WT	WT	MT	MT	WT	WT	WT	WT	WT	WT	3	39.6	29	−ve	−ve	120	1.3
8	MT	WT	WT	MT	WT	MT	WT	WT	WT	WT	WT	3	40.4	20	−ve	−ve	100	4.0
9	MT	WT	WT	MT	WT	WT	MT	WT	WT	WT	WT	3	32.3	38	−ve	−ve	200	4.0
10	MT	WT	WT	WT	WT	WT	MT	MT	WT	WT	WT	3	51.0	21	−ve	+	140	2.5
11	MT	WT	WT	MT	WT	WT	MT	WT	WT	WT	WT	3	46.5	34	+	−ve	220	3.2
12	MT	WT	WT	MT	WT	WT	WT	WT	MT	MT	MT	5	63.0	30	+++	+	180	3.0

MT: Mutant. WT: Wild Type.

### Analysis of c-Met

**Figure 2:**
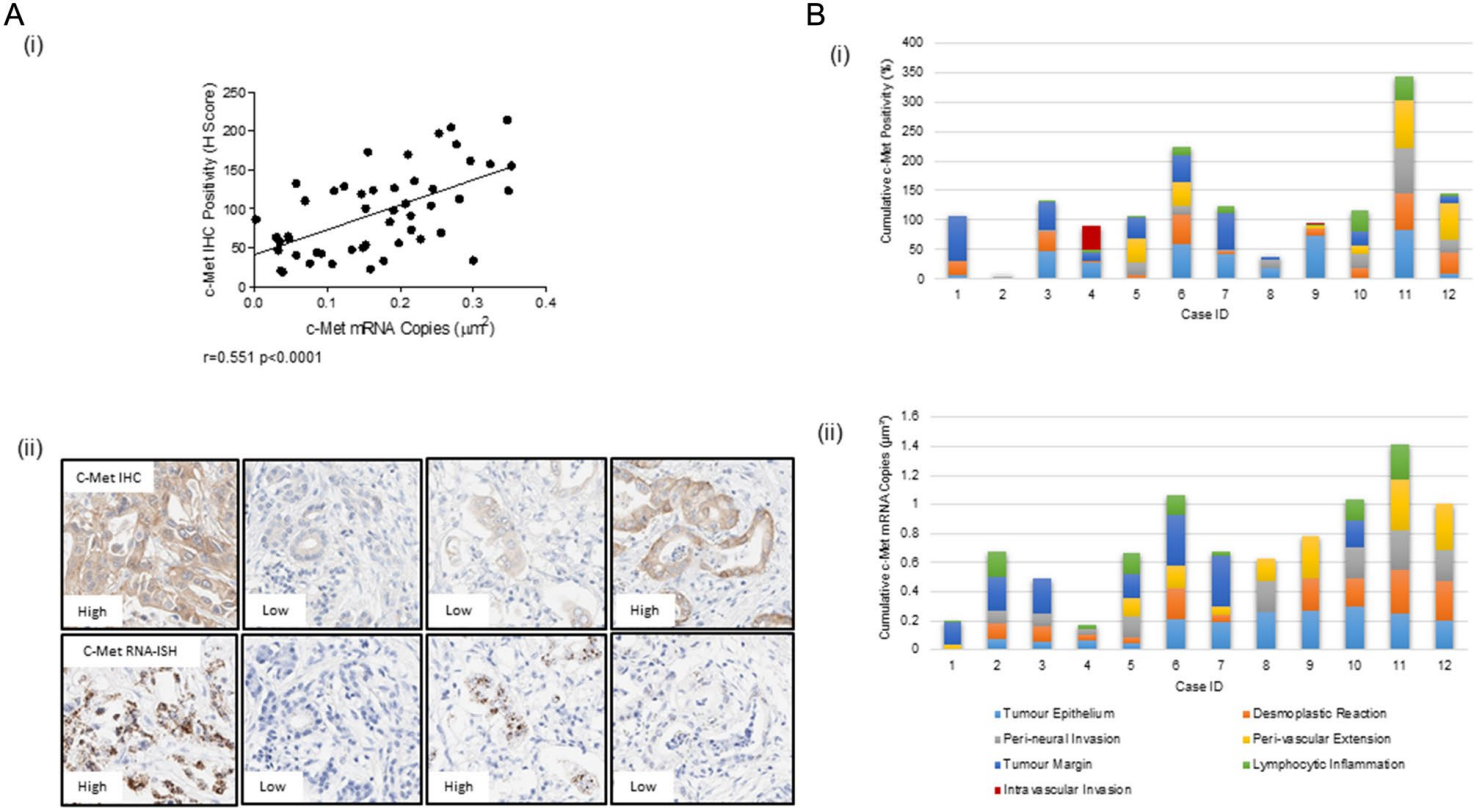
(**A**)(i) Scatter plot displaying the correlation between IHC and RNA-ISH for C-Met expression across all cores where both data were available. (ii) Representative images of concordance and discordance of c-Met staining in the two hybridisation methodologies. (**B**)(i) bar chart displaying the cumulative c-Met IHC percentage positivity for cases 1–12 encompassing the topographic areas where data was available, with c-Met RNAScope data shown in (ii).

c-Met expression, evaluated by immunohistochemistry and RNA-ISH, demonstrated positivity in the tumour epithelium of all patients (12/12). The quantitative analysis of c-Met expression showed variability by both techniques in the patient cohort. Figure 2A(i) illustrates the overall significant positive correlation between IHC compared with RNAScope (Spearman r = 0.551 p < 0.0001). However in some cases a high level of discordance across the two techniques was observed, examples of staining are shown in Fig. 2A(ii). Additionally, the expression of c-Met IHC, quantified as percentage positivity in tumour above the detection threshold determined by image analysis, in the different topographic areas of each patient was heterogeneous (Fig. 2B(i)). Likewise, the expression of c-Met RNA-ISH, quantified by probe copy number µm^2^ in tumour, also showed wide-ranging expression levels across the cases (Fig. 2B(ii)). Both techniques showed expression that predominated in the tumour rich area, followed by tumour margin, desmoplastic reaction, peri-vascular extension and peri-neural invasion, with a slightly lower expression in the lymphocytic inflammatory area. Complete data for all topographical areas was available in cases 3, 5–7 and 10–12 for c-Met IHC and case 5 for c-Met RNA-ISH.

### Mutational analysis

NGS analysis showed the presence of somatic variants in 11 of 12 PDAC cases (Table 1). The most frequent mutations were found in *KRAS, TP53* and *CDKN2A*. *KRAS* mutations were found in 10/12 of cases, *TP53* in 9/12 of cases and *CDKN2A* in 3/12 of cases; all of these were consistent with the known mutational profile of PDAC. Other somatic allelic variants were found only once and in different cases. While the numbers in this study are small to establish firm immuno-genomic correlations, interesting patterns are starting to emerge. The case with highest mutational burden (case 12), also showed high expression levels of IDO-1 (IHC) and c-Met (IHC and RNA-ISH). The top two highest scoring cases by IHC, cases 9 and 11, that also show high RNA scores, both show the same mutation pattern, having mutations in *KRAS, TP53* and *CDKN2A*. The one case that carried a mutation in the mismatch repair gene PMS2 (case 3), had the highest expression of CD3-positive T-cells. Finally, case 1, containing no detectable mutations, contained a low tumour cellularity (20%) had the lowest desmoplastic reaction, as measured by SMA, while case 12, which had the highest number of mutations, also had the most marked desmoplastic reaction. In Case 3, which had the lowest tumour content (10–15%), a *PMS2* mutation was found suggesting that the lack of *KRAS* mutation in this sample is a genuine finding. The mean target coverage in tumours for the genes on the panel was between 95 and 745, with the lowest being in Case 4, in which two mutations were detected. Cases 1 and 3, in which no *KRAS* mutations were detected, had a mean target coverage in tumours of 746 and 461, respectively which indicates that these tumours are truly wild-type and that there are no sampling or technical issues resulting in false negatives.

Mutational heterogeneity across the cases was clearly apparent. 7/12 cases displayed unique mutations. Only 3/12 cases displayed exactly the same mutations in *KRAS, TP53*, with the remaining two cases showing identical gene expression (*KRAS, TP53, CDKN2A).*

### Nanostring digital spatial profiling

**Figure 3:**
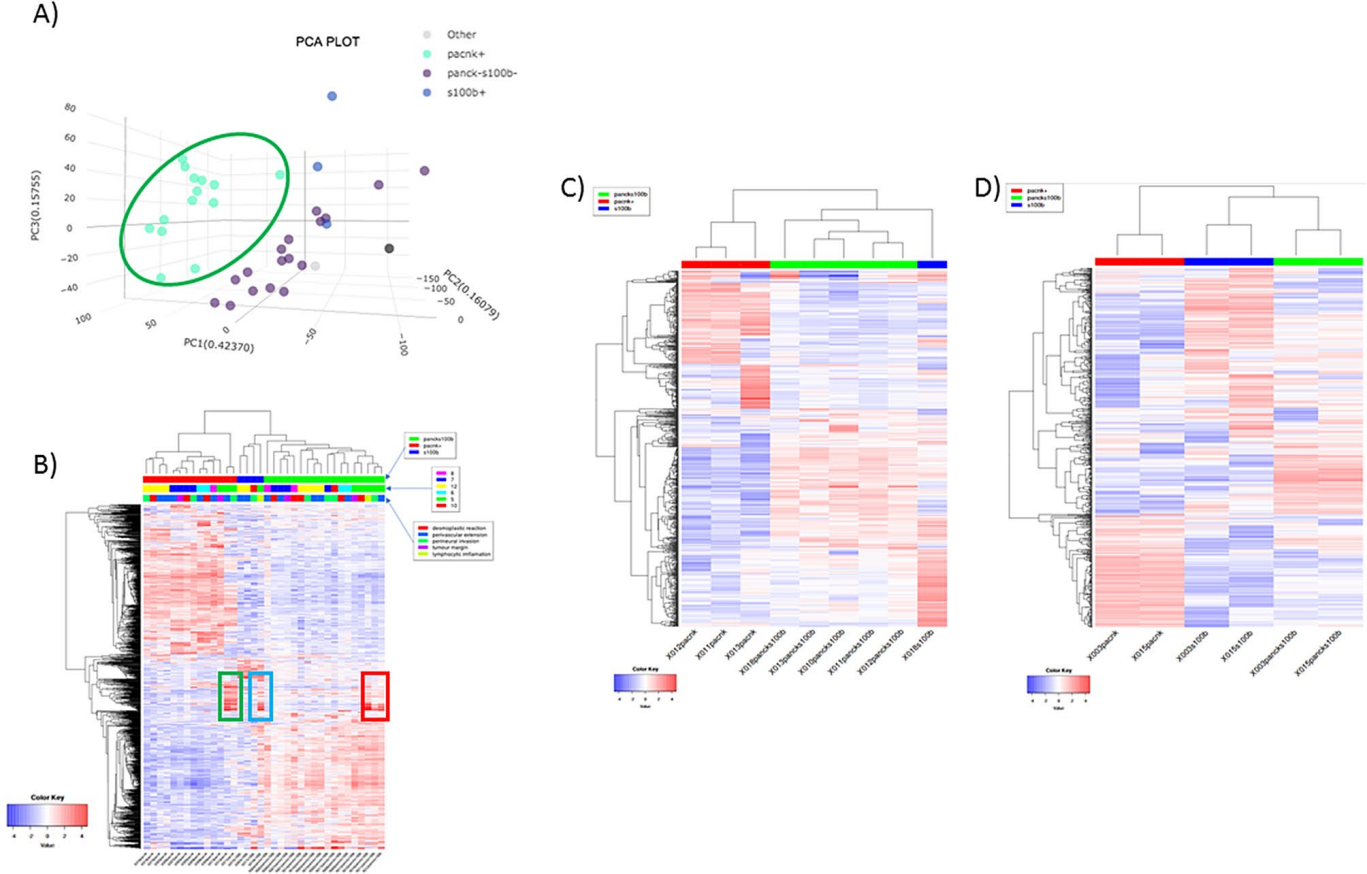
(**A**) Principle Component Analysis of the RNA data showing clustering. panCK status outlined in green. (**B**) Unsupervised hierarchical clustering of the 1000 most variable genes across all datasets clustered together based on the IHC status. Green box shows distinct panCK differences from two samples in case 5. The blue box highlights differences in the S100β+ sample from case 5, while the red box shows the difference panCK-ve/S100β− samples. (**C**) Displays the difference in heterogeneity across topograpahy within the same case (case 5), across perivascular extension, perineural invasion, and tumour margin. (**D**) Heat map showing the range of heterogeneity within the same core, where two samples were taken across the same topographical areas.

Principal Component Analysis (PCA) of the RNA data showed that samples clustered primarily on their panCK status, with panCK+ samples being clearly separate from the combined samples (Fig. 3A). These data indicate that samples from the same case show higher similarity than those from other individuals, as no clustering was observed based on topography (PCA not shown).

Unsupervised hierarchical clustering of the 1000 most variable genes across all datasets clustered together based on the IHC status (Fig. 3B). The heatmap reveals clear differentially expressed genes clustering across sample subtypes. 12 of the 14 panCK+ samples show similar expression patterns, whereas two (both from case 5) show distinct differences and cluster at the edge of the panCK+ samples (Fig. 3B, green box). Multiple samples from case 5 show upregulation of a subset of genes not seen in the other cases, with the S100β+ sample from case 5 showing distinct differences from other S100β+ samples (Fig. 3B, blue box), and likewise seen in panCK-ve/S100β− samples (Fig. 3B, red box).

Case 5 evidences precisely the range of heterogeneity that may be present within different topographical cores from the same case. panCK+ regions from three different topographic cores from case number 5 showed a clear split into a pair of similar expression patterns (perivascular extension and perineural invasion), and one (tumour margin) that showed a markedly different expression pattern (Fig. 3C). When these samples are seen in the context of all samples tested, the tumour margin sample is more similar to the panCK+ expression patterns seen in other cases and topographical cores, with the perivascular extension and perineural invasion samples being situated on a different branch of the cluster tree (Fig. 3B).

K-means clustering of gene expression patterns across case 5 split in to six clear gene ontology biological processes (Table 2). Unsurprisingly, perivascular extension and perineural invasion samples showed higher expression of genes involved in complement cascade, cell junction organisation, post-translational protein phosphorylation, interleukin-10 signalling, platelet degranulation and chemokine-chemokine receptor pathways (Table 2 Cluster C). Interestingly, these samples also showed higher expression of genes involved in pancreatic secretion, protein and fat digestion and absorption (Table 2 Cluster E).

**Table 2:** K-means clustering of gene expression patterns displayed by gene ontology biological process.

Cluster	adj.Pval	nGenes	Pathways	Genes
A	5.36E−52	74	Extracellular matrix organization	COL11A1 COL16A1 ADAMTS2 MMP2 MMP11 COL1A1 MMP19 COL10A1 COL5A1 POSTN LOXL2 COL4A2 SULF1 COL8A1 SERPINH1 PDPN COL1A2 COL3A1 COL4A1 COL4A5 COL5A2 COL15A1 FAP AEBP1 TPSAB1 ELN FBLN1 CCDC80 CRISPLD2 COMP ENG NID1 CCN2 FMOD PDGFRA CYP1B1 EMILIN1 LUM FKBP10 CCN1 SFRP2 DDR2 OLFML2B ANTXR1 EFEMP2 LCP1 GREM1 SH3PXD2B COL12A1 ITGAL DCN VCAN NID2 TIMP1 SERPINE1 SH3PXD2A SPARC FN1 MFAP2 LRP1 MATN3 CTSL ITGA11 COL6A1 COL6A2 CTSK ADAM12 ITGB2 COL6A3 HTRA1 FBN1 A2M BGN PECAM1
A	6.07E−29	92	Cell adhesion	MYL9 NID2 PTK7 FN1 CCN2 PLAU PALLD COL5A1 POSTN PLXNC1 GPNMB PARVG CDH11 CCN1 THY1 VSIG4 SPON2 ITGB2 PDPN OLR1 ITGBL1 PECAM1 SPON1 SNAI2 FBLN1 COL16A1 SERPINE1 SPOCK1 IGFBP7 FBN1 CERCAM COL3A1 ANTXR1 LEP RAP2B LILRB4 HLA-DRB1 HLA-DRA AIF1 ITGAL STAB1 CD4 CD74 VCAN SPI1 FAP PTPRC CCDC80 LGALS1 CORO1A COMP ENG COL1A1 NRXN2 COL12A1 NID1 CSF3R LRP1 EMILIN2 LRRC32 ITGA11 CYP1B1 EMILIN1 COL6A1 COL6A2 COL8A1 BOC ADAM12 SPARCL1 MXRA8 COL6A3 GREM1 AXL IL7R HLA-DQB1 NTM THBS2 COL15A1 DUSP1 PDGFRA HLA-DPA1 EFEMP2 HLA-DPB1 HLA-DMB SFRP1 SFRP2 LAPTM5 CCL19 ARHGDIB ISLR LOXL2 DDR2
A	6.57E−29	32	Collagen fibril organization	COL5A1 LOXL2 SERPINH1 AEBP1 COL11A1 ADAMTS2 MMP11 COMP FMOD CYP1B1 EMILIN1 LUM FKBP10 SFRP2 DDR2 COL5A2 COL1A1 COL1A2 GREM1 COL3A1 EFEMP2 COL12A1 COL16A1 COL10A1 COL4A2 COL6A1 COL6A2 COL8A1 COL6A3 COL4A1 COL4A5 COL15A1
A	2.66E−24	60	Blood vessel development	ADGRA2 ISM1 ENG COL1A1 CALD1 ANGPTL2 THY1 THBS2 COL15A1 HSPB6 DCN SPARC NR4A1 SERPINF1 COL4A2 SULF1 LEP SPI1 FAP MMP2 COMP PTK7 FN1 PRRX1 CCN2 TBX2 MMP19 APOE COL5A1 PDGFRA TBX3 CYP1B1 EMILIN1 FKBP10 CCN1 COL8A1 SFRP2 EDNRA FGF18 ITGB2 FMNL3 CYBB GREM1 ANTXR1 SOCS3 COL4A1 C5AR1 SERPINE1 ACTA2 PECAM1 STAB1 PTGIS LOXL2 ADAM12 COL1A2 SFRP1 PDGFRB LRP1 EFEMP2 GPNMB
A	3.75E−24	61	Vasculature development	ADGRA2 ISM1 ENG COL1A1 CALD1 ANGPTL2 THY1 THBS2 COL15A1 HSPB6 DCN SPARC NR4A1 SERPINF1 COL4A2 SULF1 LEP SPI1 FAP MMP2 COMP PTK7 FN1 PRRX1 CCN2 TBX2 MMP19 APOE COL5A1 PDGFRA TBX3 CYP1B1 EMILIN1 FKBP10 CCN1 COL8A1 SFRP2 EDNRA FGF18 ITGB2 FMNL3 CYBB GREM1 ANTXR1 SOCS3 COL4A1 C5AR1 SERPINE1 ACTA2 PECAM1 STAB1 PTGIS LOXL2 ADAM12 COL1A2 SFRP1 PDGFRB LRP1 PDPN EFEMP2 GPNMB
A	2.72E−22	84	Cell migration	CORO1A ENG PDGFRB PLXNC1 THY1 FGF18 FCER1G ITGB2 FMNL3 PDPN AXL GP2 SDC2 CCL19 CD248 RAP2B ITGBL1 CCL18 ITGAL DCN VCAN FBLN1 FAP SFRP1 SERPINE1 COL1A1 ARHGDIB SPARC FN1 IGFBP5 PLAU NR4A1 APOE PDGFRA GPNMB CCN1 AIF1 PECAM1 CD74 ADGRA2 SPI1 TREM2 IL1R1 CCN2 CSF3R EPX LRP1 PALLD SERPINF1 POSTN CYP1B1 EMILIN1 TRPM2 SFRP2 CXCL14 CYGB DDR2 FSTL1 CTHRC1 GREM1 LDB2 LEP F2R SLC8A1 C5AR1 SNAI2 PTPRC TIMP1 RARRES2 PTK7 DUSP1 COL5A1 LOXL2 LCP1 SULF1 ITGA11 NKD1 SPOCK1 COL3A1 ZEB2 TREM1 JCHAIN COL1A2 OLR1
A	1.02E−21	71	Circulatory system development	ADGRA2 ISM1 ENG COL1A1 FN1 CALD1 TBX3 ANGPTL2 THY1 PDLIM3 THBS2 COL15A1 ELN HSPB6 DCN SPARC NR4A1 SERPINF1 COL4A2 SULF1 LEP COL11A1 SPI1 FAP MMP2 COMP PTK7 PRRX1 ID3 CCN2 TBX2 MMP19 APOE COL5A1 PDGFRA CYP1B1 EMILIN1 FKBP10 CCN1 COL8A1 SFRP2 EDNRA FGF18 ITGB2 FMNL3 CYBB GREM1 ANTXR1 SH3PXD2B SOCS3 COL4A1 C5AR1 SERPINE1 ACTA2 ADAP2 PECAM1 STAB1 PTGIS LOXL2 ADAM12 COL1A2 FBN1 COL3A1 SNAI2 SFRP1 PDGFRB LRP1 PDPN EFEMP2 SLC8A1 GPNMB
A	5.07E−21	87	Cell motility	CORO1A ENG PDGFRB PLXNC1 THY1 FGF18 FCER1G ITGB2 FMNL3 PDPN AXL GP2 SDC2 CCL19 CD248 RAP2B CFAP43 ITGBL1 CCL18 ITGAL DCN VCAN FBLN1 FAP SFRP1 SERPINE1 COL1A1 ARHGDIB SPARC FN1 IGFBP5 PLAU NR4A1 APOE PDGFRA GPNMB CCN1 AIF1 PECAM1 CD74 ADGRA2 SPI1 TREM2 IL1R1 CCN2 CSF3R EPX LRP1 PALLD SERPINF1 POSTN CYP1B1 EMILIN1 TRPM2 SFRP2 CXCL14 CYGB DDR2 FSTL1 CTHRC1 GREM1 LDB2 LEP CAVIN1 F2R SLC8A1 C5AR1 SNAI2 PTPRC TIMP1 RARRES2 PTK7 DUSP1 COL5A1 LOXL2 LCP1 SULF1 ITGA11 NKD1 SPOCK1 SLC9B1 COL3A1 ZEB2 TREM1 JCHAIN COL1A2 OLR1
A	9.64E−20	51	Blood vessel morphogenesis	ADGRA2 ISM1 ENG CALD1 ANGPTL2 THY1 THBS2 COL15A1 HSPB6 DCN SPARC NR4A1 SERPINF1 COL4A2 SULF1 LEP SPI1 FAP MMP2 COMP FN1 PRRX1 CCN2 TBX2 MMP19 APOE PDGFRA CYP1B1 EMILIN1 FKBP10 CCN1 COL8A1 SFRP2 EDNRA FGF18 ITGB2 FMNL3 CYBB GREM1 COL4A1 C5AR1 SERPINE1 STAB1 PTGIS LOXL2 ADAM12 SFRP1 PDGFRB LRP1 EFEMP2 GPNMB
A	1.63E−19	90	Locomotion	CORO1A RARRES2 ENG PDGFRB PALLD PLXNC1 THY1 FGF18 FCER1G ITGB2 FMNL3 PDPN AXL GP2 SDC2 CCL19 CD248 RAP2B CFAP43 ITGBL1 CCL18 ITGAL DCN VCAN FBLN1 FAP SFRP1 SERPINE1 COL1A1 ARHGDIB SPARC FN1 IGFBP5 PLAU NR4A1 APOE PDGFRA GPNMB CCN1 AIF1 PECAM1 CD74 ADGRA2 SPI1 TREM2 PTK7 IL1R1 CCN2 CSF3R EPX LRP1 LSP1 SERPINF1 POSTN CYP1B1 EMILIN1 TRPM2 BOC SFRP2 CXCL14 CYGB DDR2 FSTL1 CTHRC1 GREM1 LDB2 LEP CAVIN1 F2R SLC8A1 FPR3 C5AR1 SNAI2 PTPRC TIMP1 DUSP1 COL5A1 LOXL2 LCP1 SULF1 ITGA11 NKD1 SPOCK1 SLC9B1 COL3A1 ZEB2 TREM1 JCHAIN COL1A2 OLR1
A	1.11E−18	110	Anatomical structure morphogenesis	GAS7 TYROBP ADGRA2 LZTS1 ISM1 ENG COL12A1 MFAP2 DUSP1 CALD1 PALLD PDGFRA TBX3 PLXNC1 ANGPTL2 PARVG CDH11 THY1 FGF18 FMNL3 PDPN FBN1 GREM1 SDC2 NPTX1 FAM20C THBS2 COL15A1 ELN HSPB6 DCN FBLN1 SERPINE1 SPARC FN1 INHBA NR4A1 COL5A1 SERPINF1 COL4A2 SULF1 CCN1 ANTXR1 LEP COL5A2 COL11A1 SPI1 FAP SLC1A3 MMP2 TREM2 ACP5 CORO1A SFRP1 COMP ASPN LIPA COL1A1 PTK7 PDGFRB IGFBP5 PRRX1 ID3 CCN2 CSF3R TGFB3 TBX2 MMP19 LRP1 APOE POSTN CYP1B1 EMILIN1 NKD1 FKBP10 CD53 COL8A1 BOC SFRP2 SERPINH1 EDNRA ITGB2 CTHRC1 CYBB HTRA1 AXL IL7R CD248 TMEM119 SOCS3 COL4A1 C5AR1 MAFB LST1 COL6A1 STAB1 CRISPLD2 SH3PXD2A PTGIS LOXL2 ADAM12 COL1A2 SNAI2 ACTA2 ACTG2 EFEMP2 GPNMB DKK3 MGP F2R
B	0.000131348	66	System process	GABRA3 GLRA2 CRYBA1 CHRNE NMU TNNI3 SCN7A SCN11A OR5B2 OR5AP2 OR13G1 GAL C1QTNF3 SLIT2 HAND2 LPO KCNMB2 LAMC3 CALCRL TRPM3 SOX10 ZMYND8 AVP TRPA1 CLIC5 SLC8A2 NPY DBH CD36 HTR2B CCN3 SNCA NCAM2 S100B SELENON TMEM100 BEST1 BFSP2 OR4D5 KCND3 OR52W1 GPR4 SLC17A8 NRXN1 SLC9A4 OR4F6 RTL4 OR51I2 FZD9 RELN OR51A2 OPN1MW3 OR52R1 OR8S1 RANGRF KLF2 TFAP2A CACNA2D1 MAPT ANK3 AKAP6 SLC16A1 SORBS1 MEOX2 OTOR HOXB2
B	0.000212035	47	Nervous system process	GABRA3 GLRA2 CRYBA1 CHRNE SCN7A SCN11A OR5B2 OR5AP2 OR13G1 LPO KCNMB2 LAMC3 TRPM3 SOX10 ZMYND8 AVP TRPA1 NMU CLIC5 SLC8A2 DBH TNNI3 CD36 CCN3 SNCA NCAM2 S100B TMEM100 BEST1 BFSP2 OR4D5 OR52W1 SLC17A8 NRXN1 OR4F6 RTL4 OR51I2 FZD9 RELN OR51A2 OPN1MW3 OR52R1 OR8S1 TFAP2A MAPT ANK3 OTOR
B	0.005891948	57	Movement of cell or subcellular component	SEMA3B LAMC3 FGF20 SOX10 CCL1 TSPAN11 KIF17 EPHA7 KIF20B MYO1F SLIT2 CFAP157 SDC3 CXCR1 GAP43 CCR9 CLEC14A FAM110C RELN LAMA2 THBS4 CCN3 IGFBP3 DLC1 RET HOXB9 NDNF CSF1 APOD L1CAM RASGEF1A NGFR MMP9 LAMA4 DBH PLP1 HTR2B GDF7 GFRA3 TRIM46 HAND2 SHH KCND3 ROBO2 MEOX2 NAV3 SH3BP1 ZMYND8 RANGRF CACNA2D1 MAPT GTSE1 F10 TNNI3 SHC3 SLC16A1 NRXN1
B	0.005891948	11	Regulation of nervous system process	SOX10 ZMYND8 AVP NMU SLC8A2 CCN3 S100B TMEM100 SCN11A NRXN1 RELN
B	0.005891948	20	Regulation of membrane potential	GABRA3 GLRA2 CHRNE SCN7A SCN11A AKAP6 TCL1A RANGRF KCND3 KCNMB2 ZMYND8 SLC8A2 CD36 SNCA NRXN1 RELN CACNA2D1 MAPT ANK3 FZD9
B	0.005891948	22	Axon development	SEMA3B PLP1 EPHA7 SLIT2 GAP43 LAMA2 ROBO2 MAPT L1CAM NGFR GDF7 GFRA3 NCAM2 TRIM46 SHH RET RELN ANK3 APOD SHC3 S100B NRXN1
B	0.00628404	51	Locomotion	SEMA3B LAMC3 FGF20 SOX10 CCL1 TSPAN11 EPHA7 SLIT2 CFAP157 SDC3 CXCR1 GAP43 CCR9 CLEC14A FAM110C RELN LAMA2 THBS4 CCN3 IGFBP3 DLC1 RET HOXB9 NDNF CSF1 ROBO2 APOD L1CAM RASGEF1A NGFR MMP9 LAMA4 DBH PLP1 HTR2B KIF20B GDF7 SNCA GFRA3 TRIM46 HAND2 SHH MEOX2 NAV3 SH3BP1 ZMYND8 GTSE1 F10 SHC3 SLC16A1 NRXN1
B	0.006555771	10	Action potential	SCN7A SCN11A AKAP6 KCNMB2 SLC8A2 CD36 KCND3 RANGRF CACNA2D1 ANK3
B	0.006555771	15	Axon guidance	SEMA3B EPHA7 SLIT2 GAP43 LAMA2 L1CAM NGFR GDF7 GFRA3 SHH ROBO2 RELN SHC3 RET NRXN1
B	0.006924573	39	G protein-coupled receptor signaling pathway	GABRA3 CALCRL GAL GLRA2 CCL1 NMU ADGRG6 GCG HCRTR1 NPY HTR2B GPR119 OR5B2 OR5AP2 GPR4 OPN1MW3 FCN1 SNCA MARCO CXCR1 NPY5R OR4D5 CCR9 OR52W1 BHLHA15 GPR132 OR4F6 OR51I2 FZD9 OR13G1 OR51A2 DGKK OR52R1 OR8S1 GPHA2 AVP PDE1A ARHGEF6 GAP43
B	0.007856876	13	Glial cell differentiation	ADGRG6 PLP1 DAAM2 SHH LAMC3 SOX10 LGI4 S100B TAL1 GAP43 RELN LAMA2 MAPT
B	0.009205009	42	Cell migration	SEMA3B LAMC3 FGF20 SOX10 CCL1 TSPAN11 SDC3 CXCR1 CCR9 CLEC14A FAM110C RELN THBS4 CCN3 SLIT2 IGFBP3 DLC1 RET HOXB9 NDNF CSF1 APOD L1CAM RASGEF1A MMP9 LAMA4 DBH PLP1 HTR2B KIF20B GFRA3 TRIM46 HAND2 SHH LAMA2 MEOX2 NAV3 SH3BP1 ZMYND8 GTSE1 F10 SLC16A1
B	0.009462674	5	Response to pain	TRPA1 THBS4 DBH RET RELN
B	0.009658947	6	Autonomic nervous system development	SOX10 GFRA3 HAND2 RET HOXB2 TFAP2A
C	1.36E−09	16	Humoral immune response	CXCL2 CXCL6 C3 CRP S100A9 CXCL3 CXCL1 CXCL8 C4B SERPING1 DEFB1 FGA CLU CFB CD81 CCL2
C	7.00E−06	30	Defense response	CXCL2 CCL2 CXCL6 CRP S100A9 CXCL3 CXCL1 DEFB1 CXCL8 UBD THBS1 SERPING1 FGA ICAM1 CD81 SPP1 CLU C3 CHI3L1 GJA1 SERPINA1 CFB C4B MMP12 IL32 SCTR NCF2 DCDC2 NCAM1 FLNA
C	1.23E−05	29	Response to oxygen-containing compound	CXCL2 GRB10 CXCL6 LYPD1 S100A9 CXCL3 CXCL1 CXCL8 CFTR ICAM1 CCL2 AKAP7 SPP1 THBS1 ATP1A1 TNC HOMER2 SLC39A14 CRYAB SOD2 TNFSF10 GJA1 CLDN3 AGRN HNF1B TPM1 CLU FLNA MMP12
C	1.32E−05	42	Response to organic substance	FSTL3 CXCL2 GRB10 CCL2 PMEPA1 CXCL6 LYPD1 S100A9 CXCL3 CXCL1 CXCL8 UBD LEFTY1 CFTR ICAM1 AKAP7 SPP1 CLU THBS1 ATP1A1 C4B TNC RAP1GAP HOMER2 SLC39A14 CRYAB CD81 SOD2 TNFSF10 SNRPN CHI3L1 GJA1 CLDN3 FLRT2 AGRN MMP12 HNF1B MT1G SLC34A2 FLNA IL32 NCAM1
C	1.32E−05	9	Antimicrobial humoral response	CXCL2 CXCL6 S100A9 CXCL3 CXCL1 CXCL8 DEFB1 FGA CLU
C	1.92E−05	6	Humoral immune response mediated by circulating immunoglobulin	CRP CLU C3 SERPING1 C4B CD81
C	1.92E−05	7	Complement activation	C3 CRP C4B SERPING1 CLU CFB CD81
C	1.92E−05	25	Cell adhesion	FGA FLNA ICAM1 CDH6 SPP1 CLDN10 S100A9 CLDN3 CLDN2 FSTL3 CD81 THBS1 CXCL8 PPP1CB MMP12 TNC CNN3 SDC4 AFDN NCAM1 FLRT2 CCL2 TPM1 PKHD1 IL32
C	1.92E−05	38	Response to external stimulus	CXCL2 CCL2 CXCL6 MTUS1 CRP S100A9 CXCL3 CXCL1 DEFB1 CXCL8 FGA UBD SPP1 THBS1 SERPING1 C4B TNC ICAM1 CD81 SOD2 CLU C3 NFIB GJA1 ATP1A1 FLRT2 FLNA CFB MMP12 CHI3L1 TUBB2B CLDN3 SCTR NCAM1 PPP1CB RAP1GAP GRB10 NCF2
C	1.92E−05	14	Regulation of peptidase activity	SERPINA4 HIP1R SERPING1 SERPINA5 SERPINA1 TNFSF10 THBS1 S100A9 TFPI2 CRYAB ITIH5 C3 C4B CLDN3
C	1.92E−05	7	Antimicrobial humoral immune response mediated by antimicrobial peptide	CXCL2 CXCL6 S100A9 CXCL3 CXCL1 CXCL8 DEFB1
C	1.92E−05	46	Regulation of biological quality	FGA FLNA SLC4A4 SLC39A14 KDELR1 SPP1 MT1G SLC34A2 ATP1A1 WNK2 MT1E MT1F AKAP7 CCL2 CLU C3 HIP1R CRP THBS1 CFTR WWTR1 ICAM1 HOMER2 TFPI2 GRB10 SOD2 RBP1 CXCL6 SERPING1 GJA1 GATM FLRT2 AGRN SERPINA1 GTF2I HNF1B CRYAB CD81 TPM1 CLDN3 PKHD1 SCTR ACSM3 NCF2 S100A9 SERPINA5
C	2.75E−05	31	Movement of cell or subcellular component	CXCL2 CCL2 SDC4 CXCL6 MYO1B MTUS1 KIF12 TUBB2B DCDC2 S100A9 CXCL3 CXCL1 CXCL8 FLNA THBS1 MMP12 ICAM1 GRB10 CD81 NFIB ATP1A1 FLRT2 SOD2 AFDN TPM1 GJA1 DEFB1 CLDN3 NCAM1 PKHD1 RAP1GAP
C	2.75E−05	16	Response to bacterium	CXCL2 CXCL6 S100A9 CXCL3 CXCL1 DEFB1 CXCL8 CCL2 FGA C4B ICAM1 SOD2 C3 NFIB GJA1 CRP
D	3.99E−10	4	Cell wall disruption in other organism	REG1A REG3G REG3A REG1B
D	1.09E−08	7	Digestion	CEL PRSS3 CLPS CTRB1 CTRB2 PRSS2 SERPINA3
D	1.09E−08	7	Antimicrobial humoral response	REG1A REG3G REG3A REG1B PLA2G1B PRSS3 PRSS2
D	4.07E−07	13	Proteolysis	PRSS3 CPA1 CELA3A CPB1 CPA2 CTRC CTRB1 CTRB2 SERPINA3 CELA3B PRSS2 SPINK1 INS
D	4.66E−07	4	Cobalamin metabolic process	PRSS3 CTRC CTRB1 CTRB2
D	6.99E−07	5	Antimicrobial humoral immune response mediated by antimicrobial peptide	REG1A REG3G REG3A REG1B PLA2G1B
D	8.63E−06	4	Acute-phase response	INS REG3G REG3A SERPINA3
D	0.000216407	6	Response to peptide hormone	REG1A REG3G REG3A REG1B INS PLA2G1B
D	0.000243582	9	Response to external biotic stimulus	REG1A REG3G REG3A REG1B PLA2G1B INS CLPS PRSS3 PRSS2
E	2.11E−06	6	Maintenance of gastrointestinal epithelium	VSIG1 TFF3 TFF2 TFF1 MUC4 MUC13
E	4.43E−06	28	Response to external biotic stimulus	GSDMB OAS1 PI3 TRIM31 S100A14 APOL1 CD55 UPK1B REG4 TRIM29 PLAC8 HPGD GJB2 IFI27 C15ORF48 FOS PLAAT3 KRT16 FER1L6 CEACAM1 PPARG MUC5B XAF1 MUC4 MUC17 MUC3A MUC13 MUC5AC
E	8.23E−06	7	O-glycan processing	MUC5B GCNT3 MUC4 MUC17 MUC3A MUC13 MUC5AC
E	1.81E−05	9	Digestion	VSIG1 TFF3 TFF2 TFF1 CAPN9 GCNT3 CAPN8 MUC4 MUC13
E	3.22E−05	39	Response to external stimulus	LAMA3 GSDMB OAS1 PI3 PPARG TRIM31 XCR1 S100A14 F3 APOL1 CD55 WNT11 UPK1B SLC2A1 REG4 TRIM29 PLAC8 HPGD TNFRSF11B GJB2 IFI27 AVPR1A C15ORF48 FOS KRT20 PLAAT3 NQO1 KRT16 FER1L6 MMP28 CEACAM1 PLAT MUC5B XAF1 MUC4 MUC17 MUC3A MUC13 MUC5AC
E	0.000206501	31	Biological process involved in interspecies interaction between organisms	GSDMB OAS1 PI3 TRIM31 S100A14 APOL1 TMPRSS4 CD55 UPK1B REG4 TRIM29 PLAC8 CREB3L1 HPGD GJB2 IFI27 C15ORF48 FOS PLAAT3 KRT16 HSPA1A FER1L6 CEACAM1 PPARG MUC5B XAF1 MUC4 MUC17 MUC3A MUC13 MUC5AC
E	0.000250704	7	Digestive system process	VSIG1 TFF3 TFF2 TFF1 GCNT3 MUC4 MUC13
E	0.000257976	14	Multicellular organismal homeostasis	VSIG1 TFF3 TFF2 TFF1 PLAC8 TNFRSF11B SFN KRT16 USH1C OAS1 MUC4 MUC13 SLC2A1 ID1
E	0.000270783	17	Epithelial cell differentiation	USH1C UPK1B SPRR1B VSIG1 AGR2 SDC1 ID1 SPRR3 SFN KRT16 WNT11 CEACAM1 PODXL PPARG DHRS9 PI3 KRT20
E	0.000302632	10	Tissue homeostasis	VSIG1 TFF3 TFF2 TFF1 TNFRSF11B USH1C OAS1 MUC4 MUC13 SLC2A1
E	0.000455402	20	Defense response to other organism	GSDMB OAS1 PI3 TRIM31 APOL1 TRIM29 PLAC8 IFI27 KRT16 CD55 S100A14 CEACAM1 PPARG MUC5B XAF1 MUC4 MUC17 MUC3A MUC13 MUC5AC
F	2.42E−18	52	Cell adhesion	CDH1 DSG2 NTN4 DSP ITGB8 PERP ITGB6 LAMA5 ITGB4 PTPRF NECTIN4 TNFRSF21 CCL28 PCDH1 CLDN1 PLXNB1 ADAM9 CLDN7 PKP3 CLDN4 PLXNB2 LAMB3 ERBB3 CD46 EPCAM EPHA2 CXADR F11R SPINT2 EFNA1 JUP MUC1 ADGRG1 CD24 ITGA3 BAIAP2L1 LAMC2 KRT18 SOX9 ATP1B1 NFKBIZ MPZL2 PTPRK ITGA2 EZR TESC LIF ASS1 DDR1 FUT3 TACSTD2 CD151
F	2.42E−18	62	Tissue development	LAMC2 NTN4 SOX9 LAMA5 ST14 F11R SEMA4B LAMB3 DSP LIF DAB2IP CD151 AKR1C1 EZR HNF4A KLF5 KRT18 TFCP2L1 ITGB6 DHCR24 IRF6 PPL EPCAM KRT17 RAB25 EHF FLNB SHROOM3 DUOX2 EPHA2 CLDN1 ELF3 ITGA2 SPINT1 SPINT2 EFNA1 FAM83H PLXNB2 ITGA3 MMP15 VIL1 ITGB4 ADAM9 AKR1C3 ANXA4 PROM1 DSG2 MYH14 ITGB8 DDR1 CD24 ESRP1 CXADR TACSTD2 KRT6A KRT23 PERP KRT7 KRT8 KRT19 JUP PKP3
F	1.07E−17	39	Epithelial cell differentiation	ST14 F11R DSP LIF AKR1C1 EZR HNF4A KLF5 KRT18 TFCP2L1 IRF6 PPL SOX9 KRT17 RAB25 EHF FLNB SHROOM3 EPHA2 CLDN1 ELF3 ITGA2 SPINT2 VIL1 ADAM9 AKR1C3 ANXA4 PROM1 CD24 ESRP1 DSG2 KRT23 PERP KRT7 KRT8 KRT19 JUP PKP3 KRT6A
F	1.10E−16	29	Skin development	DSP ST14 LSR ITGB6 DHCR24 IRF6 PPL SOX9 KRT17 LAMA5 FLNB CLDN1 ITGA2 JUP ADAM9 AKR1C3 ITGA3 ITGB4 EPHA2 CLDN4 DSG2 KRT23 KRT18 PERP KRT7 KRT8 KRT19 PKP3 KRT6A
F	2.99E−16	47	Epithelium development	SOX9 LAMA5 ST14 F11R DSP LIF CD151 AKR1C1 NTN4 EZR HNF4A KLF5 KRT18 TFCP2L1 IRF6 PPL EPCAM KRT17 RAB25 EHF FLNB SHROOM3 EPHA2 CLDN1 ELF3 ITGA2 SPINT1 SPINT2 PLXNB2 VIL1 ADAM9 AKR1C3 ANXA4 PROM1 DDR1 CD24 ESRP1 TACSTD2 KRT6A DSG2 KRT23 PERP KRT7 KRT8 KRT19 JUP PKP3
F	2.40E−12	47	Cell motility	NTN4 ITGB8 ITGB6 LAMA5 ITGB4 CCL28 CXCL5 PLXNB1 CD151 FAM83H SEMA4B PLXNB2 LAMB3 VIL1 MMP7 ANXA3 DUOXA2 GRB7 PTPRK F11R SPINT2 ADAM9 EFNA1 ADGRG1 ITGA3 EPCAM SOX9 DAB2IP EPHA2 ITGA2 DUOX2 JUP CDH1 LAMC2 SLC12A2 RAB25 DDR1 PTPRF CXADR CLDN1 FUT3 CLDN4 SMIM22 TACSTD2 CD24 ERBB3 ATP1B1
F	2.49E−12	61	Anatomical structure morphogenesis	PROM1 LAMC2 NTN4 SOX9 VIL1 LAMA5 EPHA2 PLXNB1 EFNA1 CDC42EP4 SEMA4B PLXNB2 LAMB3 LIF DAB2IP ANXA3 F11R KRT19 CD151 ADGRG1 EZR DSP HNF4A KLF5 MYH14 SHB PERP TFCP2L1 ITGB6 KRT17 LDLR ITGB4 RAB25 FLNB SHROOM3 DUOX2 ST14 ELF3 ITGA2 SPINT1 SPINT2 KRT8 SPTBN2 TNFAIP2 ITGA3 MMP15 JUP PKM ITGB8 DDR1 NBEAL2 ADAM9 CLDN4 SLC12A2 TACSTD2 KRT6A ABLIM1 KRT18 IER3 GRB7 MPZL2
F	5.59E−12	53	Movement of cell or subcellular component	LAMC2 NTN4 ITGB8 ITGB6 MYO5C LAMA5 ITGB4 EPHA2 CCL28 CXCL5 PLXNB1 EFNA1 CD151 FAM83H SEMA4B PLXNB2 LAMB3 VIL1 MMP7 ANXA3 DUOXA2 GRB7 PTPRK F11R SPINT2 ADAM9 ADGRG1 ITGA3 MYH14 EPCAM SOX9 DAB2IP ITGA2 DUOX2 JUP CDH1 DSG2 SLC12A2 DSP RAB25 DDR1 PTPRF CXADR CLDN1 FUT3 CLDN4 SMIM22 TACSTD2 CD24 ERBB3 EZR ATP1B1 SPTBN2
F	6.64E−12	49	Locomotion	LAMC2 NTN4 ITGB8 ITGB6 LAMA5 ITGB4 EPHA2 CCL28 CXCL5 PLXNB1 EFNA1 CD151 FAM83H SEMA4B PLXNB2 LAMB3 VIL1 MMP7 ANXA3 DUOXA2 GRB7 PTPRK F11R SPINT2 ADAM9 ADGRG1 ITGA3 EPCAM SOX9 DAB2IP ITGA2 DUOX2 JUP CDH1 SLC12A2 RAB25 DDR1 PTPRF CXADR CLDN1 FUT3 CLDN4 SMIM22 TACSTD2 CD24 ERBB3 EZR ATP1B1 SPTBN2
F	1.04E−11	36	Regulation of cellular component movement	PLXNB1 FAM83H SEMA4B PLXNB2 VIL1 MMP7 ANXA3 DUOXA2 GRB7 CCL28 PTPRK SPINT2 ADAM9 CD151 ITGA3 EPCAM SOX9 LAMA5 DAB2IP EPHA2 ITGA2 EFNA1 ADGRG1 DUOX2 JUP CDH1 DSG2 LAMC2 DSP RAB25 CLDN1 FUT3 CLDN4 SMIM22 TACSTD2 ERBB3
F	1.22E−11	43	Cell migration	NTN4 ITGB8 ITGB6 LAMA5 ITGB4 CCL28 CXCL5 PLXNB1 CD151 FAM83H SEMA4B PLXNB2 LAMB3 VIL1 MMP7 ANXA3 GRB7 PTPRK F11R ADAM9 EFNA1 ADGRG1 ITGA3 SOX9 DAB2IP EPHA2 ITGA2 JUP CDH1 LAMC2 SLC12A2 RAB25 DDR1 PTPRF CXADR CLDN1 FUT3 CLDN4 SMIM22 TACSTD2 CD24 EPCAM ATP1B1
F	2.73E−11	34	Regulation of cell motility	PLXNB1 FAM83H SEMA4B PLXNB2 VIL1 MMP7 ANXA3 DUOXA2 GRB7 CCL28 PTPRK SPINT2 ADAM9 CD151 ITGA3 EPCAM SOX9 LAMA5 DAB2IP EPHA2 ITGA2 EFNA1 ADGRG1 DUOX2 JUP CDH1 LAMC2 RAB25 CLDN1 FUT3 CLDN4 SMIM22 TACSTD2 ERBB3
F	3.44E−11	14	Cornification	DSG2 DSP KRT23 KRT18 PERP PPL KRT17 KRT7 ST14 KRT8 KRT19 JUP PKP3 KRT6A

In addition to the heterogeneity across topographical cores from the same case there is also evidence of heterogeneity within individual cores, as shown in the peri-vascular extension core of case 12. Two distinct but matching AOIs were interrogated within this core, each sample covering panCK+, panCK-ve/S100β− and S100β+. Whilst clustering shows that the IHC subsets do cluster more closely than samples from the individual regions of the core (Fig. 3D), the correlation in gene expression between the IHC subtypes is lower than would have been expected. Particularly for the S100β+ samples (r = 0.483), indicating that although the two S100β+ sites sampled are within the same TMA core, they show a sizeable degree of heterogeneity just a few micrometers from one another.

## Discussion

The nature and dynamics of PDAC immune composition is largely unknown but is presumed to be influenced by factors such as desmoplasia^27^. Therefore, it would appear that a detailed analysis of biomarkers related to specific pathophysiological hallmarks may be essential to understand biomarker heterogeneity and complexity. Herein, we introduce the concept of *Topographic TMAs* as a technical platform to interrogate the heterogeneity of cancer pathophysiology, towards the characterisation of a genuine tumour microenvironment. We demonstrate that such a design is feasible and can be verified with a small set of well characterised and targeted immunohistochemical biomarkers. Other studies that have utilised TMAs in the context of PDAC have adopted a more limited approach whereby cores are only described as derived from tumour or from the invasive margin but without detailed information on histological selection^28–30^. Using expert pathologist annotations (the current gold standard ground truth), we have captured specific pathophysiological hallmarks for each case.

The morphological heterogeneity of PDAC is well established in the literature, and is core to the traditional description of the disease in which most of the topographic changes are well-described^31^. Our work set out to (1) capture this morphological heterogeneity in morphology-driven TMA construction; (2) confirm this heterogeneity with markers associated with this morphology; (3) once confirmed in (1) and (2), describe the potential heterogeneity of further biomarkers that may have therapeutic implications. Accepting the heterogeneous nature of PDAC, we demonstrate that our T-TMA model is able to capture such heterogeneity. Our anticipated immunohistochemical detection of biomarkers evidences that our approach does indeed capture morphological heterogeneity.

In this proof of concept study we did not detect a relationship between T-cell infiltration and desmoplasia using CD3 and SMA as surrogates. The expression of *TGF-β* mRNA in 2 of the cases is interesting, as increased TGF-β in the tumour microenvironment represents a primary mechanism of immune evasion that promotes T-cell exclusion and blocks acquisition of the TH1-effector phenotype^30^. Immunotherapies directed against TGF-β signalling may therefore have broad applications in treating patients with advanced cancer^32^. Specifically, in early stage PDAC patients TGF-β1 overexpression has been reported to be associated with improved survival and low tumour cell proliferation^2^. We believe this is the first report to date to demonstrate TGF-β expression in PDAC by RNA-ISH technology may have a highly focal pathophysiological distribution.

IDO-1 expression was observed on tumour cells in 7/12 cases but would only have been detected in 3/12 cases if the TMA had only contained tumour rich cores. Studies on the expression of IDO-1 in PDAC are relevant as the immunosuppressive IDO pathway has been shown to be impeded in a syngeneic mouse model of PDAC using a nanocarrier approach^33^.

Quantitative c-Met expression was variable across cases and between the different topographical areas within each case, reinforcing the benefit of a topographic TMA approach to obtain a holistic perception. Pre-clinical studies suggest that the inhibition of the HGF/c-MET pathway may sensitise PDAC tumours to gemcitabine^6^. Combined approaches in orthotopic models result in a decrease in primary tumour volume and a significant reduction of metastatic tumour burden. This may represent a novel therapeutic strategy to improve outcomes in PDAC.

Mutational analysis showed that the three most frequent somatic allelic variants were in *KRAS, TP53* and *CDKN2A*. These findings, albeit in a small sample cohort, are consistent with previous genomic characterizations in PDAC^33,34^. The unique distribution of mutated genes in our panel across the cases demonstrates that, even following meticulous topographical histological selection, the underlying gene profile can have a confounding influence on the expression of the particular biomarker being investigated. We are aware of the limitations of our analysis in that this panel contains on a small portion of coding regions of the human genome and only identifies a number of key driver cancer genes. Furthermore, though our study is in line with previous genomic observations, we must resist over interpretation and generalisations within our small sample feasibility study.

Digital spatial profiling revealed gene clustering was strongly correlated based on the IHC status across all samples, suggesting tumour margins (panCK+) may be comparable across cases. However, intra-case and intra-core topographic sampling revealed broad heterogeneity with regard to biological processes, which may influence the local PDAC microenvironment. Our limited sample number precludes a robust comparison with other published PDAC gene expression profiles. However, the reliability of our analysis is buoyed by the correlation of our expression patterns in the panCK+ AOIs with that of two differential gene expression (DGE) studies on PDAC and normal tissue (data not shown)^35,36^. Direct comparison with these DGE studies is difficult as we do not compare tumour with normal samples, which may influence the expression data we see. Such a comparison would be worthy of investigation as clearly separate cell populations, such as that seen in our study, may provide a true representation of the local topographic PDAC microenvironment in comparison to studies which include both tumour and surrounding normal tissue, however carefully microdissected.

We are confident topographic TMAs can support a more holistic characterisation of genuine tumour microenvironments across many solid tumours. It is worth noting that the value of a particular representative areas may vary depending on the tissue type being evaluated. Within PDAC specifically, it could be argued that the heterogeneity associated with the desmoplastic reaction may be the most important in terms of representative area, per our described observations. Whether this representative area is the most definitive in larger cohorts remains to be seen. Cases with highest desmoplastic reaction presented the highest number of mutations, coupled with positive TGF-β expression, which itself presented with non-tumour pathophysiological distribution that may have immunotherapy treatment implications in PDAC.

The value of topographic TMAs as evidenced within our manuscript must also be caveated by the labour intensive nature of the creation of such a resource. Our approach to topographic TMAs creation was to utilise pathologist annotations, the clinical gold standard^37^. Yet, the diagnostic burden and reduction in pathologist workforce only compounds the difficulty faced when implementing our approach. However, the capabilities of deep learning offer opportunities that can be leveraged here via the algorithmic identification of representative areas to support the pathologist. The identification of varied tissue structures in PDAC H&E images by deep learning models has been proposed to support the routine diagnostic workflow^38^. Utilising proposed tissue classes presented by heat map on H&Es could accelerate representative area selection. Additionally, combining such mark-up images with the capabilities of high throughput TMA generation equipment, which supports the remote reviewing and selection of cores by an annotator, could significantly reduce the burden on the pathologist and support the implementation of this approach on larger scale TMA cohort construction.

The observations made in this study are preliminary and should be considered proof-of-principle. However, our results demonstrate the viability and strength of *Topographic TMAs* in PDAC, which comprehensively captures the extensive biomarker heterogeneity of pathophysiological regions scored digitally. This approach enriches the perception of biomarkers, with significant advantages to traditional TMAs (lacking complex representation) and full sections (where the histological hallmarks may not be present/obvious and the overall scoring can thus be misleading)^39^. That being said, TMAs have been used extensively to successfully interrogate many aspects of the tumour microenvironment for patient prognostication, by ourselves and others^16,40,41^.

In essence, in an era in which cancer heterogeneity, tissue microenvironment and tumour evolution dictates the complexity of cancer, *Topographic TMAs* may represent a *conditio sine qua non* for accurate biomarker evaluation.

### Supplementary Information

Supplementary Figure S1.

Supplementary Figure S2.

Supplementary Table S1.

## Data Availability

The data underlying the results presented in the study are available from The Precision Medicine Centre of Excellence (PMC) at Queen’s University Belfast. Contact can be made to Queen’s University Belfast, F.A.O. Professor Manuel Salto-Tellez, Health Sciences Building. 97 Lisburn Road. Belfast. BT9 7AE. Tel: + 44(0)28 9097 2293. m.salto-tellez@qub.ac.uk.
