# Supplementary figures and images for: Topographic analysis of pancreatic cancer by TMA and digital spatial profiling reveals biological complexity with potential therapeutic implications
Source: Sci Rep. 2024 May 18;14:11361. doi: 10.1038/s41598-024-62031-0 (PMC11102543; doi:10.1038/s41598-024-62031-0)

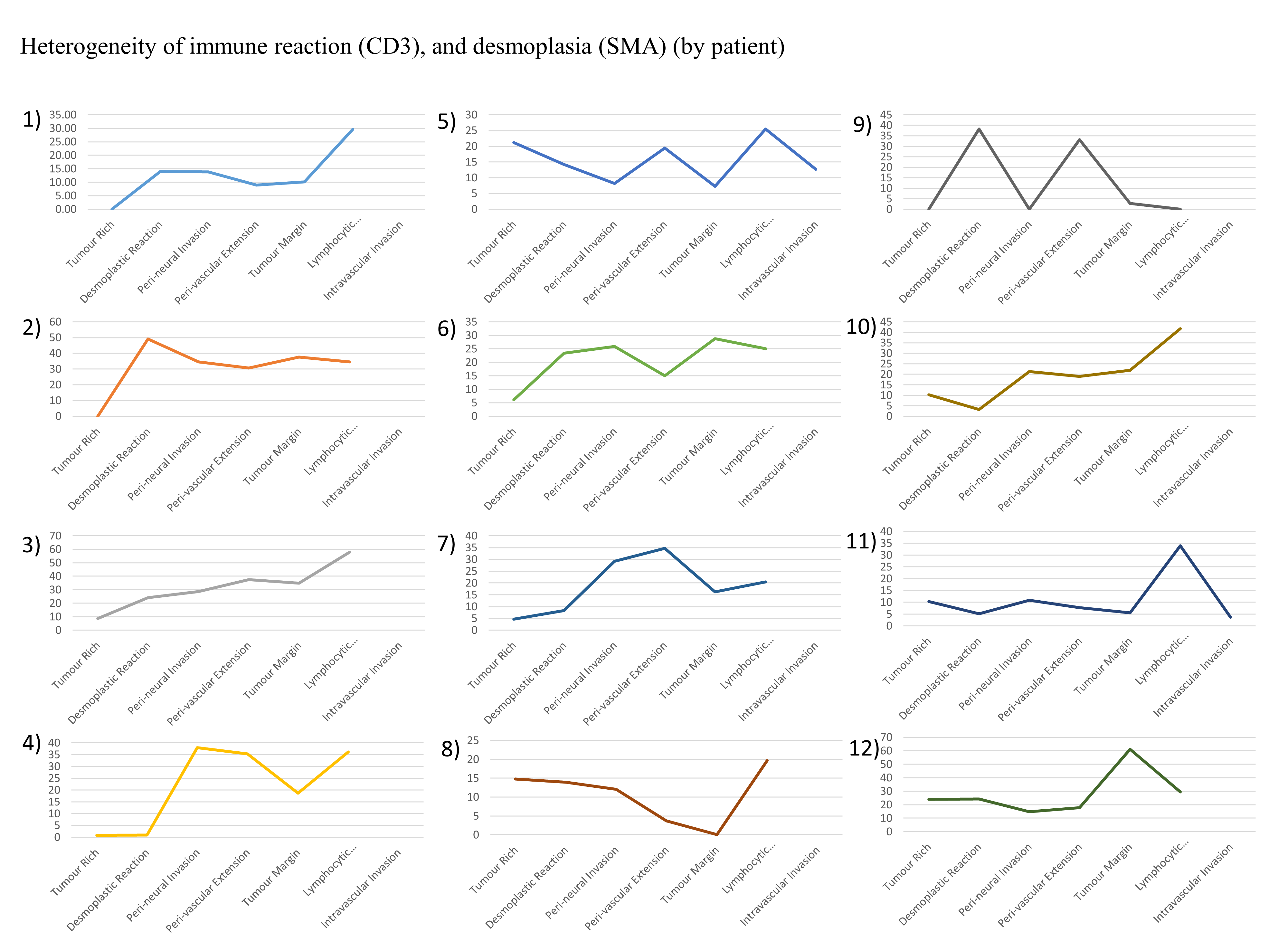

Supplement: Supplementary file 1 — Supplementary Figure S1. [file 41598_2024_62031_MOESM1_ESM.tif]

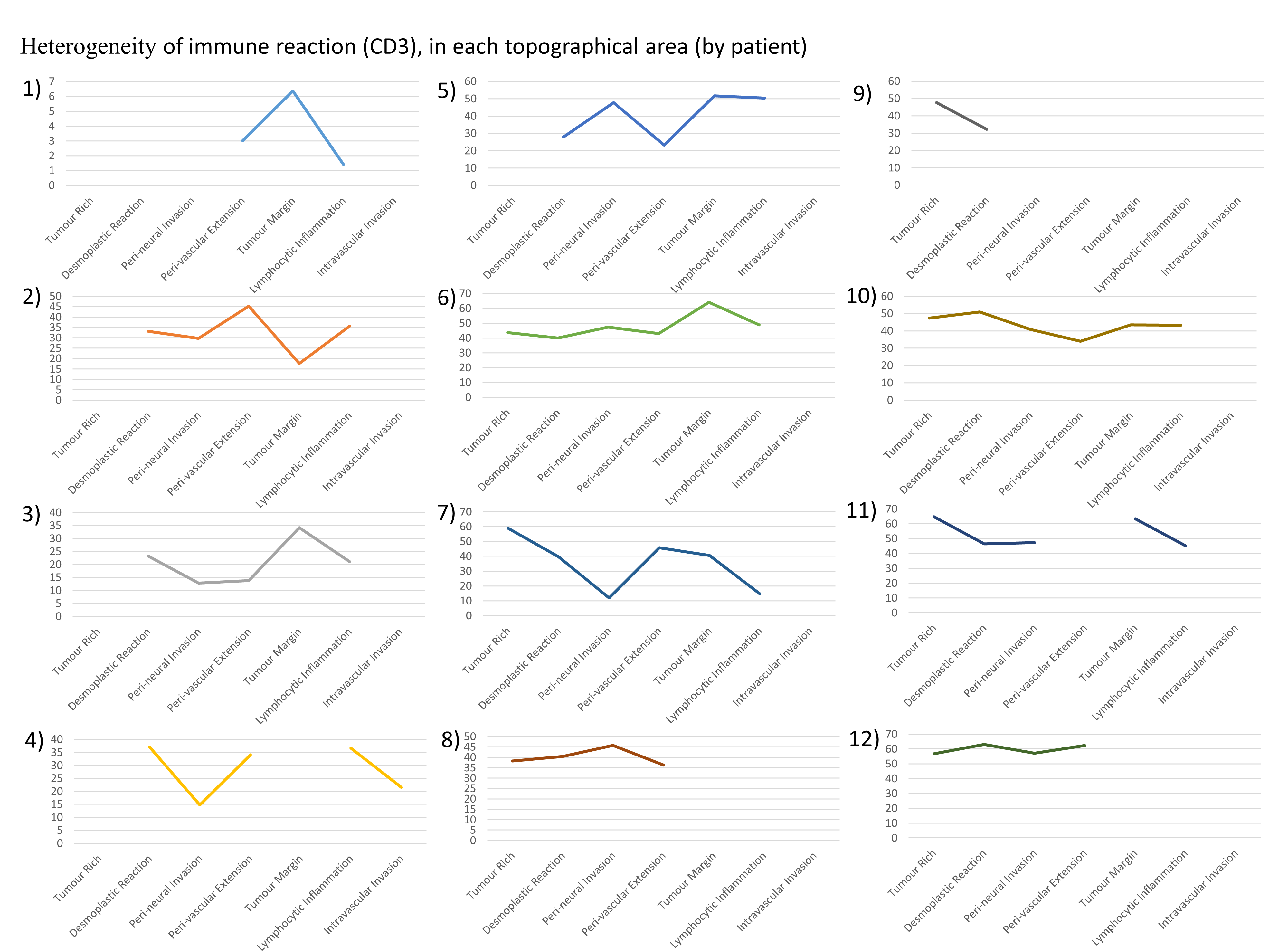

Supplement: Supplementary file 2 — Supplementary Figure S2. [file 41598_2024_62031_MOESM2_ESM.tif]
